# Multifaceted roles of CD44 in cancer progression and targeted therapeutic strategies

**DOI:** 10.1038/s12276-026-01797-x

**Published:** 2026-08-04

**Authors:** Hyun-Ji Oh, Seung-Tae Kim, Hyun-Jin Kim, Kang-To Lee, Hyungshin Yim

**Affiliations:** 1 Department of Pharmacy, College of Pharmacy, Hanyang University, Ansan, Gyeonggi-do Republic of Korea; 2 Institute of Pharmaceutical Science and Technology, Hanyang University, Ansan, Gyeonggi-do Republic of Korea

**Keywords:** Targeted therapies, Drug development

## Abstract

CD44, a multifunctional transmembrane glycoprotein, is not only a bystander but also a crucial driver of cancer progression that promotes cancer stem cell maintenance, metastasis, and resistance to therapy. Therefore, CD44 is recognized as a promising therapeutic target in advanced malignancies. Here, we discuss its unique features, such as its structural diversity, which arise from alternative splicing and the post-translational modifications of cleavage and phosphorylation. In addition, we discuss the function of CD44 as a multivalent cell adhesion receptor for extracellular matrix components, including hyaluronic acid, fibronectin, osteopontin, and TSG6, thereby regulating lymphocyte activation, cell–cell interactions, cell adhesion, and migration within the extracellular matrix. Moreover, the emerging role of CD44 as a co-receptor of receptor tyrosine kinases such as epidermal growth factor receptor, c-MET, and vascular endothelial growth factor receptor 2 is highlighted to elucidate the contribution of CD44 to malignant signaling networks. We also discuss its potential as a therapeutic target in advanced cancers, particularly its applications in unconjugated antibodies, antibody–drug conjugates, peptide-based inhibitors, and chimeric antigen receptor-T cell therapies. We conclude by addressing the limitations observed in clinical studies and outlining promising opportunities for future development.

## CD44 drives cancer progression and therapy resistance

CD44 is a transmembrane glycoprotein involved in various cellular functions, including cell adhesion, migration, and lymphocyte activation. It is recognized as a cancer stem cell marker and therapeutic target owing to its high expression in advanced cancer stages. This study explores structural diversity of CD44, focussing on its isoforms generated through alternative splicing and post-translational modifications. The authors highlight the role of CD44 in cancer progression, emphasizing its interactions with extracellular matrix components such as hyaluronic acid and osteopontin. The study reveals that glycosylation and phosphorylation of CD44 regulate its ligand-binding affinity and cell migration. Notably, interaction of CD44 with receptor tyrosine kinases, such as epidermal growth factor receptor and vascular endothelial growth factor, enhances oncogenic signaling pathways. The findings suggest that targeting CD44, particularly its variant forms, could disrupt cancer stem cell maintenance and metastasis, offering potential therapeutic avenues. This summary was initially drafted using artificial intelligence, then revised and fact-checked by the author.

## Introduction

CD44 is a transmembrane glycoprotein that functions as a co-receptor of several growth factors and cytokines. It is also known as phagocytic glycoprotein-1 in murine mesenchymal cells^1^, class III collagen receptor in human fibroblasts^2–4^, hermes antigen in human lymphocytes^5^, lymphocyte homing receptor^6^, and homing-associated cell adhesion molecule in human hematopoietic progenitors^7^. It has been identified as a multiple cell adhesion receptor for the extracellular matrix (ECM) components such as hyaluronic acid (HA)^8^, collagen^3,4^, fibronectin^3^, osteopontin^9^, and tumor necrosis factor-stimulated gene 6 (TSG6)^10^ and involved in lymphocyte activation^5^, cell–cell interactions^5^, cell adhesion^3^, and migration in the ECM.

CD44 has been studied as a cancer stem cell (CSC) marker in breast cancer, colorectal cancer, and other cancers^11,12^. Because of its high expression in several cancers, especially in advanced stages, it has been recognized as an important therapeutic target and biomarker for advanced cancer^13^. CD44 exists as a standard version and several variants formed through alternative splicing that have different expressions and functions in different tissues^13^. Owing to its extensive post-translational modification, complex structures, and diverse functions, it has been studied in the context of major diseases, especially cancer. Here, we discuss the importance of CD44 as a therapeutic target for cancer treatment, along with ways that its function depends on post-translational modifications. The cleaved CD44-mediated signaling pathway in cancer and current clinical trials using CD44 as a therapeutic target will also be discussed.

## Structural diversity of CD44 and its biological functions

### Diverse structure of CD44 and its isoforms via alternative splicing

**Fig. 1:**
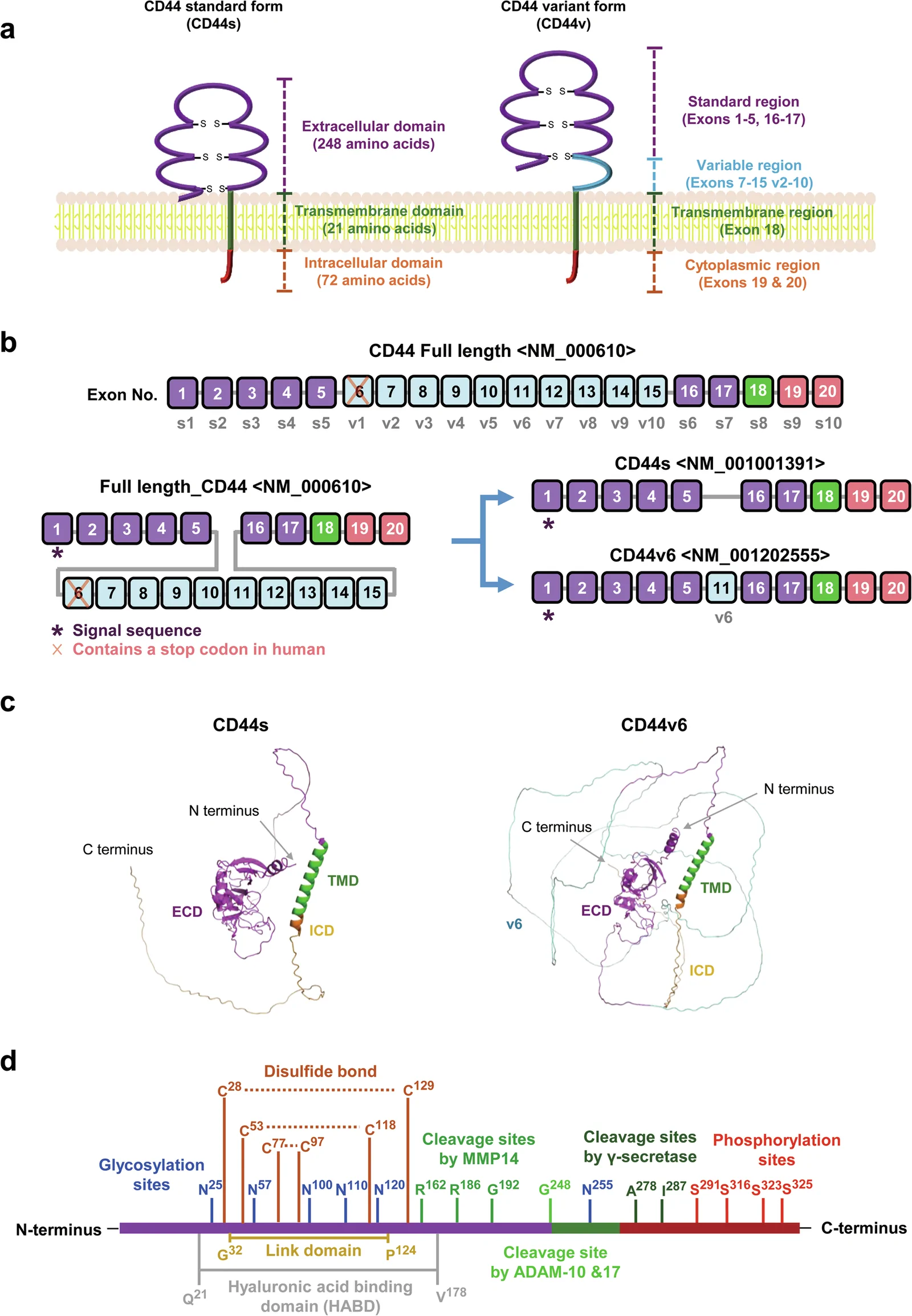
Structural and genomic organization of CD44: functional domains, alternative splicing, and post-translational modifications. **a**, Functional domains of the standard and variant forms of CD44. The CD44 protein is composed of 341 amino acids and divided into three domains: the extracellular domain (ECD) located outside the cell, the transmembrane domain (TMD) that spans the cell membrane, and the intracellular domain (ICD) located inside the cell. Each domain consists of specific exons: the ECD contains exons 1–5 and 16–17, comprising 248 amino acids; the TMD is composed of exon 18, which consists of 21 amino acids; and the ICD contains exons 19–20, consisting of 72 amino acids. CD44 isoforms are determined by the insertion of variable exons, which are located just before the TMD and are regulated through alternative splicing in humans. **b**, Exon structure of standard form and variant forms of CD44. CD44 consists of 20 exons in total. Among them, CD44s contains the 10 standard exons, and the CD44v isoforms contain the 10 standard exons and one or more of the 10 variant exons. Because the variant exon v1 contains a stop codon, it is not translated into the protein in humans, but it is translated in mice. Numerous CD44v isoforms can be generated through alternative splicing. CD44v6, one of the most representative isoforms, contains exon v6. **c**, Predicted 3D structure of CD44s and CD44v6. The protein structures of CD44s (NP_001001391.1) and CD44v6 (NP_001001389.1) were predicted using AlphaFold. As illustrated in the models, the distinct structural domains are represented in magenta (ECD region), green (TMD region), and orange (ICD region), with the v6 variant in CD44v6 highlighted in cyan. **d**, Structural domains and post-translational modifications of CD44. The hyaluronic acid-binding domain of CD44 consists of residues 21–178 and the link domain of CD44 comprises residues 32–124. Three conserved disulfide bonds exist in humans. The ECD of CD44 contains disulfide bonds formed at three specific sites. CD44 undergoes N-linked glycosylation at Asn25, 57, 100, 110, 120, and 255. Proteolytic processing occurs through matrix metalloproteinase (MMP)14 cleavage at Arg162, Arg186, and Gly192, and ADAM10/17 cleavage at Gly248, generating soluble CD44. Subsequent intramembrane cleavage by γ-secretase between Ala278 and Ile287 releases the ICD of CD44. CD44 is also phosphorylated at Ser291, Ser316, Ser323, and Ser325.

CD44 is a type I transmembrane protein that consists of an extracellular domain (ECD, 248 amino acids), a transmembrane domain (TMD) (21 amino acids), and a cytoplasmic intracellular domain (ICD, 72 amino acids)^6,14^ (Fig. 1a). Although the 341 amino acids of the standard form of CD44 (CD44s), which lacks 10 variant exons (v1–v10) (Fig. 1b,c), have a molecular weight of ~37 kDa, its apparent molecular weight is 85–95 kDa because of extensive post-translational modification, especially *N*-linked or *O*-linked glycosylation at the ECD^6,14^.

The genomic structure of CD44 reveals a notable level of complexity and diversity^15^. CD44 was found to have 19 exons in humans: 10 standard exons and 9 variable exons^15^ (Fig. 1b). Later, the CD44 exon count was extended to 20, 10 standard exons and 10 variable exons, because mouse CD44 was found to have 20 exons, with the new exon found between the standard (constant) exon 5 (s5) and variant exon 2 (v2), which is now considered to be variant exon 1 (v1)^16^. In the human CD44 gene, exon 6 (v1) was not detected in a PCR analysis of complementary DNA extracted from various tumor cell lines and cancer types because it has a stop codon at the 17th amino acid. However, it was present in mice^16^. Therefore, v1, which is absent in humans, exists in mice, so the variable exons are v1–v10 (ref. ^16^). The murine CD44 gene is known to comprise constitutive exons 1–5 and 16–20 and 10 variant exons, exons 6–15 (v1–v10). Among them, v2–v10 are expressed in humans^17^ (Fig. 1b). CD44 comprising only the constant exons is called CD44s, and CD44 with variant exons added is called variant CD44 (CD44v). The membrane-proximal ECD region of CD44 spans ~25 kb of genomic DNA and includes at least 10 alternatively spliced exons (exons 6–15; v1–10)^15^. Those variant exons (exons 6–15; v1–10) can be skipped through alternative splicing, and exon 5 also contains an alternative splice donor site^15^. Analyses of the exons encoding the cytoplasmic tails show that both the coding sequence and 3ʹ untranslated region of the long-tailed isoform are contained within exon 20 (s10)^15^, and exon 19 (s9) encodes the first three amino acids of the ICD, which are shared by both the long-tailed and short-tailed isoforms. Exon 18 (s8) encodes the TMD^15,18^ (Fig. 1a,b).

The protein structures of CD44 are variable because of the variant regions derived from v1 to v10. CD44s has exons 1–5 (s1–5) and 16–17 (s6–7) as the ECD to bind with ligands, exon 18 (s8) as the main TMD, and exons 19–20 (s9–10) as the ICD and cytoplasmic tail according to the original exon numbering of *CD44* gene (Fig. 1b,c). The domain encoding exons 1–5 at the N terminus binds with HA, a main ligand of CD44. The HA-binding domain (HABD) consists of a typical link domain of many hyaladherins and includes a shallow HA-binding groove (Fig. 1d). The link domain of murine CD44 contains two conserved disulfide bonds: Cys57–Cys123 (Cys53–Cys118 in humans) that stabilizes the core, and Cys81–Cys101 (Cys77–Cys97 in humans) at the bottom of the HA-binding groove, which stabilizes it and contacts the bound HA. A third disulfide bond, Cys32–Cys134 (Cys28–Cys129 in humans) is formed by the C-terminal and N-terminal extensions of the link domain and it stabilizes the HABD region^19^. The intracellular cytoplasmic domain is encoded by exons 19–20 and has a capacity for signaling regulation^20^.

The diversity of CD44 is generated by combinations of the 10 variant exons. CD44 containing one or more variant exons is categorized as CD44v, such as CD44v6, CD44v3–10, or CD44v8–10 (also known as CD44E). Although more than 700 different variants of CD44 can be produced theoretically^21^, only about 20 variant transcripts have been studied. Epithelial splicing regulatory protein 1 (ESRP1) and heterogeneous nuclear ribonucleoprotein M have been reported as splicing factors that regulate CD44 isoform switching in epithelial cancers including breast and prostate carcinoma^22–24^. During the epithelial–mesenchymal transition (EMT), alternative splicing of CD44 is dynamically reprogrammed^23,24^. In the early stages of the EMT, CD44v is predominant, whereas CD44s is almost exclusively present by the time the EMT is completed. In CD44 knockdown condition, re-expression of CD44s rescued the EMT, but re-expression of CD44v did not, demonstrating that CD44s is functionally essential for the EMT. During the EMT, a decrease in ESRP1 coincides with the transition from CD44v to CD44s^23^. In patient tissues, the transition from CD44v to CD44s correlates with breast cancer malignancy^23^. Reinke et al.^25^ demonstrated that the transcription factor Snail promotes the EMT by directly repressing ESRP1 via binding to the E-box in the *ESRP1* promoter. Expression of ESRP1 in immortalized human mammary epithelial cells preserved epithelial characteristics by maintaining the levels of E-cadherin and promoting the expression of CD44v. In a tamoxifen-inducible EMT model using a Snail–estrogen receptor fusion protein in human mammary epithelial cells, tamoxifen treatment activates Snail, inducing EMT and driving a CD44 isoform switch characterized by increased CD44s and decreased CD44v^25^. Therefore, the splicing pattern of newly transcribed *CD44* pre-mRNA results in the production of CD44s after the EMT, which contributes to cancer progression and phenotypic plasticity.

### Differential expression and tissue-specific distribution of CD44 isoforms in normal and malignant tissues

#### Expression and distribution of CD44 isoforms in normal tissues

CD44s is ubiquitously expressed in several normal tissues and cells, including hematopoietic cells and tissues of lung, liver, and pancreas; the variants have very limited expression in normal tissues^13^. CD44s is highly expressed in CD34^+^ hematopoietic bone marrow stem cells or progenitor cells, with average values ranging from 98.6% to 100% (ref. ^26^). Hematopoietic CD44 (CD44H), which lacks variable exons, is classified within the CD44s category^27^. By contrast, CD44v isoforms are rarely expressed in these cells. For example, the average expression levels of CD44v6 and CD44v5 range from 1.5% to 3.6% and from 0.6% to 1.4%, respectively^26^. CD44v6 expression is observed in specific epithelial tissues, including squamous epithelium (skin, cervix, and esophagus), glandular epithelium (breast and prostate), and pulmonary alveolar type II cells^28^. In quiescent hematopoietic bone marrow stem cells and progenitor cells, CD44v6 was not detected^28^.

**Fig. 2:**
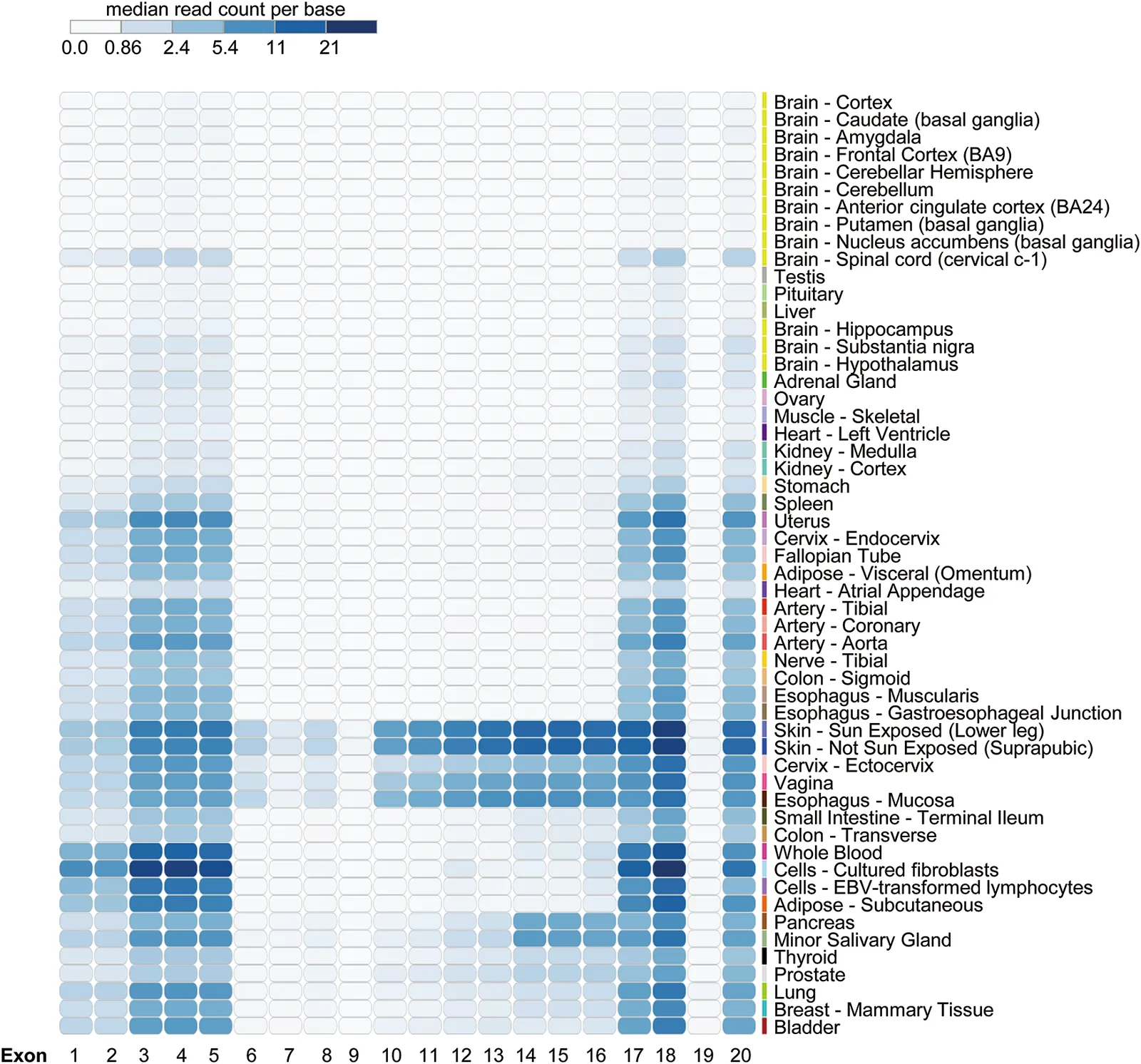
Expression profile of CD44 exons across human tissues using GTEx. The heatmap visualizes the expression profile of CD44 exons (ENSG00000026508.21) across multiple normal human tissues, using the GTEx Portal exon expression dataset (https://gtexportal.org). Columns correspond to individual exons (exons 1–20), and rows represent tissue samples from various human organs, as indicated on the right. Color intensity in each cell reflects the median read count per base for each exon–tissue pair, according to the scale bar. The heatmap allows comparison of exon-specific expression patterns across different tissues, revealing that standard exons (CD44s) are broadly expressed, whereas variant exons (CD44v) show pronounced tissue specificity, particularly in skin, cervix, vagina, and esophagus. EBV, Epstein–Barr virus.

Using the GTEx portal exon expression dataset (https://gtexportal.org), we analyzed the exon expression profiles of each exon of *CD44* and found that they differed across normal human tissues and organs (Fig. 2). In whole blood, the levels of exon 6 showed near zero intensity (Fig. 2), which supports data for a previous study^26^. Although exon 19 is a standard exon, its expression was low. Because the GTEx exon expression protocol collects reads mapped to each exon region as the median count per base, length and annotation are relatively stable, and reads accumulate well in regions such as exon 20. By contrast, exon 19 can be shorter or overlap with adjacent exons, making mapping or annotation relatively unstable. In this case, even if there is not a biologically complete absence of expression, the aggregation method can make it appear to be almost zero. According to our GTEx analysis, the standard exons were broadly expressed, but the expression of the variant exons was limited to tissues such as the skin, cervix, vagina, and esophagus (Fig. 2). Therefore, CD44s is more widely expressed than CD44v in normal tissues.

#### Expression and distribution of CD44 isoforms in malignant tissues

**Fig. 3:**
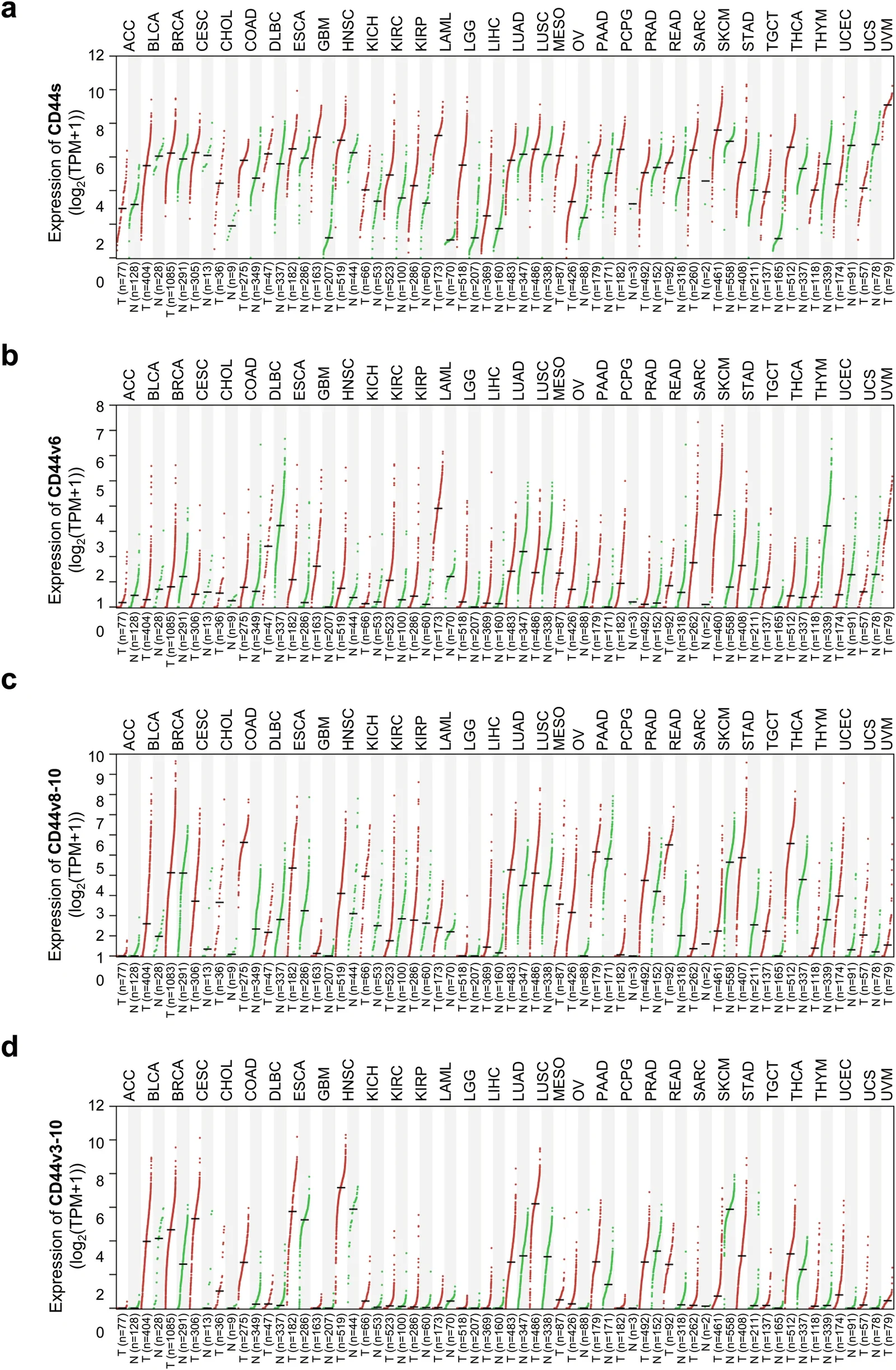
Pan-cancer analysis of CD44 isotype expression using GEPIA2. mRNA expression levels of CD44 isotypes — CD44s (part **a**), CD44v6 (part **b**), CD44v8-10 (part **c**), and CD44v3-10 (part **d**) — were analyzed across multiple cancer types using the GEPIA2 portal. Tumor tissues data (red dots) were generated from The Cancer Genome Atlas (TCGA), and normal tissues (green dots) were obtained from TCGA or GTEx. The *y*-axis represents the TPM (transcripts per million) value, and the *x*-axis indicates various cancer types. ACC, adrenocortical carcinoma; BLCA, bladder cancer; BRCA, breast invasive carcinoma; CESC, cervical squamous cell carcinoma and endocervical adenocarcinoma; CHOL, cholangiocarcinoma; COAD, colon adenocarcinoma; DLBC, diffuse large B cell lymphoma; ESCA, esophageal cancer; GBM, glioblastoma multiforme; HNSCC, head and neck squamous cell carcinoma; KICH, kidney chromophobe; KIRC, kidney renal clear cell carcinoma; KIRP, kidney renal papillary cell carcinoma; LAML, acute myeloid leukemia; LGG, brain lower grade glioma; LIHC, liver hepatocellular carcinoma; LUAD, lung adenocarcinoma; LUSC, lung squamous cell carcinoma; MESO, mesothelioma; OV, ovarian serous cystadenocarcinoma; PAAD, pancreatic adenocarcinoma; PCPG, pheochromocytoma and paraganglioma; PRAD, prostate adenocarcinoma; READ, rectum adenocarcinoma; SARC, sarcoma; SKCM, skin cutaneous melanoma; STAD, stomach adenocarcinoma; TGCT, testicular germ cell tumor; THCA, thyroid carcinoma; THYM, thymoma; UCEC, uterine corpus endometrial carcinoma; UCS, uterine carcinosarcoma; UVM, uveal melanoma.

mRNA expression levels of CD44 isotypes including CD44s, CD44v6, CD44v8-10, and CD44v3-10 were analyzed in several cancer types using GEPIA2 portal (http://gepia2.cancer-pku.cn/). Tumor tissues data were generated from The Cancer Genome Atlas (TCGA), and normal tissues were obtained from TCGA or GTEx. Our GEPIA2 analysis demonstrated that CD44s exhibits higher and more ubiquitous expression compared with CD44v isoforms across a broad spectrum of cancer types (Fig. 3).

In a meta-analysis combining data from multiple studies of carcinoma samples (total, *n* = 1,921), most primary squamous cell carcinomas expressed CD44v6 regardless of tissue origin^28,29^. Specifically, CD44v6 expression was detected in primary squamous cell carcinoma of head and neck (92.4%, *n* = 658), esophagus (96.8%, *n* = 253), lung (73.6%, *n* = 413), skin (100%, *n* = 55), cervix (58.5%, *n* = 472), and vulva (32.9%, *n* = 70), totaling 1,511/1,921 (78.7%) across cohorts. Such high expression has found in more than 90% of samples from squamous cell carcinomas of the head and neck, esophagus, and skin^28^. In adenocarcinoma of the breast (69.1%, *n* = 1,297), stomach (63.2%, *n* = 701), pancreas (64.2%, *n* = 106), and prostate (65.5%, *n* = 113), more than 50% of carcinoma cells within a given section expressed CD44v6 (ref. ^28^). Additionally, lymph node metastases associated with squamous cell carcinoma were found to express CD44v6 to the same high extent seen in the primary tumors^28^. High expression of CD44v6 in pancreatic tumor tissues was associated with increased tumor metastasis and shorter patient survival times^30^.

CD44s is highly expressed in CD34^+^ hematopoietic bone marrow stem cells and progenitor cells, and its expression was observed in CSCs^26^, which are responsible for the initiation, progression, metastasis, and recurrence of tumors. Among the various markers for CSCs, CD44 expression is distinguished in non-Hodgkin’s lymphoma and several carcinomas in breast, stomach, and colon^31–34^, indicating that it has roles in cancer development and progression. However, high surface expression of CD44v8-10 in high-grade serous ovarian cancer is associated with improved survival and an epithelial phenotype characterized by high E-cadherin and low vimentin levels^35^. In independent cohorts (*n* = 210, *P* < 0.05), median overall survival was extended by 69–73 months in patients with high expression of CD44v8-10, which was further validated by TCGA RNA-seq data (*n* = 254). Conversely, the soluble ECD of CD44v8-10, released via proteolytic cleavage, was detected in patient ascites and correlated with poor prognosis (*P* < 0.05)^35^. Therefore, CD44v8-10 exhibits a dual compartment-specific distribution, which differentially regulates EMT in high-grade serous ovarian cancer.

Collectively, CD44s ubiquitously expresses in normal tissues as well as diverse malignant tissues, whereas CD44v isoforms exhibit tissue-specific expression and functions.

### Post-translational modification of CD44 and its functions

CD44 is structurally highly heterogeneous owing to both alternative splicing of the ECD and post-translational modifications, including glycosylation, which generates a family of cell-surface glycoproteins. The changing apparent mass of CD44 is attributed to its post-translational modifications, including phosphorylation (~4 kDa)^36^, *N*-linked glycosylation (~18 kDa)^37^, and *O*-linked glycosylation (~2 kDa)^38^. The main post-translational modifications of CD44 are *N*-linked and *O*-linked glycosylation at the ECD and phosphorylation at the cytoplasmic tail. Cleavage of CD44 also supports additional functions and signals.

#### Glycosylation

*N*-linked glycosylation, a process that transfers the oligosaccharide moiety of a lipid-linked oligosaccharide to the asparagine residue of a protein, mainly occurs at the consensus sequence Asn-X-Thr/Ser (X ≠ Pro) of CD44s^37^ (Fig. 1d). To examine how altered glycosylation influences CD44-mediated HA binding, specific *N*-glycosylation sites of CD44 were mutated and global glycosylation was inhibited^38^. Those results showed that both the integrity of specific *N*-linked glycosylation sites and the membrane-proximal Ser–Gly motifs are required for CD44-HA binding, indicating that *N*-linked glycosylation of CD44 is important for its ligand interaction and function.

It has also been reported that N-glycosylation at Asn25/57/100 near the link domain of CD44 regulates the binding affinity of HA, a main ligand of CD44, whereas *N*-glycosylation at Asn110/120/255, which is further away from the link domain, regulates CD44 aggregation^38,39^ (Fig. 1d). Those results suggest a dynamic regulatory mechanism between low-affinity rolling and high-affinity firm adhesion states, depending on the glycosylation pattern. Using mutated CD44s at *N*-glycosylation sites, it was demonstrated that glycosylation deficiency prevents cell membrane transport and reduces mobility. In addition, a site-directed mutagenesis analysis identified Asn57/100/110 as key glycosylation sites essential for membrane transport and the promotion of migration^40^. Moreover, glycosylation deficiency led to endoplasmic reticulum stress induction and accelerated protein degradation^40^. Therefore, *N*-glycosylation of CD44 acts as a switch for ligand binding affinity and functions as a sophisticated regulator of motility and metastasis.

*O*-glycosylation is initiated by the transfer of a single GalNAc residue to Ser or Thr via *N*-acetylgalactosaminyltransferase, after which the GalNAc can be further extended or modified^41^. The CD44v isoform containing v3–v10 was found to carry substantially more *O*-linked glycans than CD44s, as evidenced by its pronounced reduction in molecular weight after treatment with neuraminidase and *O*-glycanase^41,42^. Prior studies have demonstrated that proteoglycan-type CD44 variants that include v3 exhibit markedly diminished HA-binding capacity, compared with CD44s, suggesting that alternative splicing of v3, together with its associated glycosylation, can attenuate CD44–HA interactions^42^. Additional work demonstrated that CD44H (lacking v8–v10) binds HA far more effectively than CD44E (containing v8–v10). These findings indicate that variants incorporating the v8–v10 exons undergo *O*-glycosylation that disrupts and suppresses CD44-HA binding^41^.

#### Phosphorylation

It has been reported that CD44 undergoes extensive phosphorylation at the ICD (290–361 amino acid residues of CD44s) of the cytoplasmic tail^4,43^. Three phosphorylation sites in the ICD of CD44s (Ser325, Ser316, and Ser291) have been studied intensively (Fig. 1d and Table 1) and found to be integral to multiple cellular regulatory processes^44–47^. CD44H has several serine residues within its ICD that are highly conserved across species, including Ser316, Ser323, and Ser325 (Fig. 1d). Analyses of mutants in which those residues were replaced with small neutral amino acids revealed that phosphorylation at Ser323 and Ser325 was minimal or undetectable. Using the mutants, Neame and Isacke^43^ revealed that both Ser325 (main phosphorylation site) and Ser323, but not Ser316, are required for phosphorylation in cells, but they do not regulate membrane localization or cytoskeletal interaction. The functions and kinases of CD44 phosphorylation at Ser325 were revealed 10 years later^44^. The Isacke group found that CaMKII (Ca^2+^/calmodulin-dependent protein kinase) phosphorylates CD44 at Ser325 (ref. ^44^). They observed that phosphorylation of CD44 at Ser325 increases when active CaMKII is overexpressed. In addition, increased CaMKII activity upregulates the phosphorylation of CD44 at Ser325. When the phosphorylation ability was eliminated by using a non-phosphomimetic mutant at Ser325, HA binding efficiency remained unchanged, but the HA-dependent cell migration function is lost^44^. These findings provide important evidence that the ligand binding and migration ability of CD44 are regulated at separate levels.

**Table 1:** Phosphorylation sites and associated kinases regulating CD44 functions.

Phosphorylation residue	Kinase	Function	Refs.
Ser325	CaMKII	p-Ser325-CD44 functions CD44-dependent cell migration on a hyaluronan substratum	^43,44,151^
Ser323	–	p-Ser323-CD44 is required for the CD44 phosphorylation in cells	^43^
Ser316	PKA (protein kinase A) and PKC (protein kinase C)	p-Ser316-CD44 functions directional sensing and chemotaxis	^45^
Ser291	PKC	p-Ser291-CD44 modulates the interaction between CD44 and ezrin, thereby regulating CD44-dependent directional cell motility	^46^
		p-Ser291-CD44 inhibits FBXO21-mediated degradation of MDR1 and consequently blocks MDR1-mediated drug resistance	^47^

CD44 also has a role in directional sensing and chemotaxis. A phosphorylation-deficient Ser316 mutant impaired CD44-dependent directional responses to a phorbol ester gradient^45^, although the mutation did not affect cell motility or directional migration to chemoattracts. Phosphorylation of CD44 at Ser316 arose after treatment with phorbol ester and the activation of protein kinase C (PKC) and protein kinase A (PKA)^45^. Ser316 of CD44 can be directly phosphorylated by PKA in vitro as well^45^. It was reported that PKC phosphorylates CD44 at Ser291 to modulate the interaction between CD44 and the cytoskeletal linker protein ezrin, which is important for CD44-dependent directional cell migration^46^. In cell migration, phosphorylation at Ser291 exerts an effect opposite to that of Ser325, indicating a reciprocal regulatory relationship between these two sites^44,46^. Notably, phosphorylation of CD44 at Ser291 inhibits the FBXO21-directed degradation of multidrug resistance 1 (MDR1) encoded by *ABCB1* (ref. ^47^). MDR1 is a major ATP-dependent efflux pump and transporter of broad drug substrates. Phosphorylation of CD44 at Ser291 increases the levels of MDR1, thereby contributing to MDR1-mediated drug resistance. Collectively, CD44 phosphorylation regulates ligand-binding affinity, directional sensing, cell migration, and chemoresistance.

#### Cleavage

CD44 sequentially undergoes proteolytic cleavage at specific sites within its ectodomain, generating either a soluble CD44 (sCD44) ectodomain or the ICD of CD44. Initially, CD44 is cleaved at the extracellular juxtamembrane region by matrix metalloproteases (MMPs) such as MMP14 or MMP9 at the Arg162–Thr163, Arg186–Ser187, and Gly192–Tyr193 bonds on the cell surface^48–51^ (Fig. 1d and Table 2). This cleavage releases sCD44 into the ECM^20,48,49^. Inhibition of MMP14 activity using synthetic MMP inhibitor BB94 and endogenous MMP inhibitors TIMP2 or TIMP6 has been shown to suppress CD44 cleavage and reduce the levels of sCD44 in melanoma cells^52^.

**Table 2:** Proteolytic cleavage of CD44 and resulting fragments.

Enzyme	Function	Cleaved residue	Cleavage product	Refs.
MMP9	Functions as an extracellular matrix (ECM)-degrading metalloprotease; mediates CD44 ectodomain shedding	Extracellular juxtamembrane region	Soluble CD44 (sCD44) ectodomain and C-terminal fragment	^50,51^
MMP14	Cleaves CD44H during cell retraction and invasion to promote cell motility	Arg162, Arg186, and Gly192	sCD44 ectodomain and C-terminal fragment	^48,49^
ADAM10	Acts as a major constitutive CD44 sheddase; enhanced by growth factor or calcium stimulation	Gly248	sCD44 and C-terminal fragment	^54,55,152^
ADAM17	Functions as an inducible CD44 sheddase; contributing to inflammatory immune cell migration	Membrane-proximal ectodomain	sCD44 and C-terminal fragment	^53^
Meprin β	Serves as a cell‑surface sheddase that cleaves CD44 ectodomain and modulates downstream protease network	Extracellular juxtamembrane region	37 kDa C-terminal fragment	^57^
γ-secretase	Operates as an intramembrane protease that releases CD44‑intracellular domain (ICD) for transcriptional regulation	Ala278, Ile287	CD44-ICD	^58,59^

In addition, ADAM10 and ADAM17 regulate CD44 ectodomain cleavage in response to distinct signaling cues. ADAM10 is activated by Ca^2+^ influx through dissociation with calmodulin, whereas ADAM17 is activated by PKC and small GTPase Rac in response to phorbol ester stimulation. The activated ADAM10 or ADAM17 induces proteolytic cleavage of CD44 (ref. ^53^). Treatment of ADAM-specific inhibitor GW280264X suppressed CD44 cleavage and reduced sCD44 levels. Silencing ADAM10 with small interfering RNA (siRNA) markedly decreased the levels of sCD44, whereas depletion of ADAM17 or MMP14 exhibited weaker effects than that of ADAM10 in human melanoma cells^52,54,55^. Mass spectrometry analysis after treatment with aspartic protease identified an ADAM10 cleavage site on CD44s at Gly248–Ser249 site^54,55^, supporting the role of ADAM10 as a constitutive sheddase of CD44 in melanoma. In head and neck squamous cell carcinoma (HNSCC), ADAM17 is recognized as a major metalloprotease, demonstrated by loss-of-function experiments using chemical inhibitors or short hairpin RNA (shRNA)^56^. Furthermore, the metalloproteinase meprin β also contributes to CD44 ectodomain cleavage. Meprin β, a cell-surface sheddase, cleaves the CD44 ectodomain, producing an ~37 kDa C-terminal fragment in HeLa cells^57^. Collectively, the dominant sheddase differs depending on the cell types and cellular context.

Inside the cell, the presenilin/γ-secretase complex cleaves the ICD region of CD44 (refs. ^58,59^), releasing the ICD fragment (CD44-ICD). The CD44-ICD translocates from the cytoplasm to the nucleus, where it regulates the expression of target genes and performs transcriptional activation functions^20^. In glioblastoma, after MMP9 cleaves CD44, γ-secretase (presenilin-dependent complex) cleaved at the bond of Ala278–Leu279 and Ile287–Ala288 as a secondary cleavage, releasing CD44-ICD. In lung adenocarcinoma (LUAD), treatment with TSG6, a HA-binding protein, increases MMP9 expression and that leads to an increase in CD44-ICD expression. The upregulated CD44-ICD then enters the nucleus and forms a complex with AP-1 and Smad2/3, thereby contributing to the EMT and tumor-associated macrophage polarization^60^. Therefore, CD44 undergoes sequential proteolytic cleavage by γ-secretase and metalloproteases including MMP14, MMP9, ADAM10, and ADAM17, which generates its functional fragments that regulate transcription for malignant progression.

## Biological functions of CD44

### Ligands of CD44

The N-terminal ECD of CD44 binds with ligands of the ECM components such as HA^8^, osteopontin^61^, collagen^3,4,62^, TSG6 (ref. ^63^), fibronectin^3,62^, and laminin^62^. These interactions contribute to several events including lymphocyte activation^5^, cell–cell interactions^5^, cell adhesion^3^, and migration within the ECM.

#### Hyaluronic acid

HA, a key component of the ECM that regulates normal structural integrity and development, modulates tissue responses during injury, recovery, and regeneration^64^. CD44 was first identified as a receptor for HA in a 1990 study using a CD44–IgG fusion protein, which investigated whether CD44 binds HA in tissue sections and lymph node high endothelial venules. The results showed that CD44 binding to high endothelial venules was sensitive to hyaluronate-degrading enzyme. This interaction disappeared after hyaluronidase treatment, providing the first evidence that CD44 binds to HA^8^. Structurally, it has reported that the HABD of CD44 consists of residues 21–178, with the core HA-binding region located between Gly32 and Pro124. This region corresponds to the link domain (residues 32–124), which has a central role in HA interaction^65^ (Fig. 1d). A cross-saturation analysis of NMR experiments revealed that 12 amino acid residues — Arg29, Lys38, Arg41, Tyr42, Lys68, Arg78, Tyr79, Asn100, Asn101, Tyr105, Arg150, and Arg154 — directly contact HA and are critical for binding^65^. In addition, the affinity of CD44 for HA is modulated by N-glycosylation, which suggests that CD44 is a dynamic regulator between low-affinity rolling and high-affinity firm adhesion states depending on its glycosylation pattern. Therefore, CD44 functions not merely as a receptor but as a sophisticated regulator of inflammatory leukocyte homing and tumor metastasis^39^.

Beyond simple binding, molecular dynamics simulations revealed that the HA–CD44 interaction proceeds through three distinct binding modes, unlike the single strong binding mode observed in X-ray crystallography. In the initial stage of their interaction, CD44 predominantly binds to HA through the weaker binding modes (modes 2 and 3). These weak interactions allow CD44 to undergo 1D diffusion along the HA polymer, thereby accelerating receptor aggregation. These multiple weak binding modes facilitate diffusion and clustering^66^. Therefore, these multiple weak interactions enhance the efficiency of physiological processes such as leukocyte rolling, adhesion, cell aggregation, and various inflammatory responses.

Functionally, HA contributes to inflammation and cancer progression depending on its molecular size and cellular contexts. High molecular weight of HA maintains tissue integrity and homeostasis, whereas, in tumor, it promotes cancer progression through the interaction with CD44. In the case of low molecular weight of HA, it acts as a pro-inflammatory signal, inducing cytokine production and chronic inflammation. Receptor for hyaluronan-mediated motility (RHAMM) cooperates with CD44 to enhance HA-driven cell motility and invasiveness, thereby promoting tumor progression^67^. The dual functions of CD44 and the cancer-specific role of CD44v6 including cooperation with receptor tyrosine kinases (RTKs), clustering with RTKs, and survival signaling amplification highlight the need for context-dependent therapeutic targeting^67^. HA–CD44v interactions maximize resistance to apoptosis and MDR1 expression by simultaneously activating RTKs including ErbB2, epidermal growth factor receptor (EGFR), and c-MET within lipid rafts and a PI3K–Akt–β-catenin–COX2-positive feedback loop. Furthermore, RHAMM, a glycosylphosphatidylinositol-anchored protein, regulates HA-mediated migration and ERK-MAPK signaling together with CD44 (ref. ^67^). Taken together, these findings indicate that CD44 acts as a dynamic regulator of inflammation and malignancy, underscoring the importance of context-dependent therapeutic targeting of the HA–CD44 axis.

#### Osteopontin

Osteopontin is an extracellular matrix protein and multifunctional glycoprotein involved in diverse physiological and pathological processes^68^. The interaction between CD44 and osteopontin has been analyzed using flow cytometry, fluorescence microscopy, and GST-pull-down assay, revealing a concentration-dependent association^9^. Osteopontin deficiency led to decreased CD44 surface expression in osteoclasts derived from mice. Treatment with osteopontin restored CD44 expression in the osteopontin-deficient cells^61^. Therefore, CD44 and osteopontin interact and are mutually regulated.

In the perivascular niche of glioblastoma, osteopontin and CD44 induce the CSC phenotype through spatial colocalization. When osteopontin binds to CD44 or CD44v6, it generates CD44-ICD through γ-secretase-mediated proteolytic cleavage, which subsequently translocates to the nucleus and activates HIF-2α via the CBP/p300 co-activator^69^. HIF-2α enhances the transcription of hypoxia-responsive genes (for example, *VEGF*) and stemness genes (for example, *SOX2* and *NANOG*), leading to the expression of DNA repair genes and anti-apoptotic factors that confer radioresistance^69^. Consistently, CD44 expression in patients with human glioblastoma correlates with the hypoxia-induced gene signature and serves as an independent negative prognostic factor for overall survival^69^.

In CD44^+^ colorectal cancer, osteopontin highly released from TAMs binds to CD44, which enhances tumorigenicity through JNK signaling^70^. Tissue microarray using osteopontin^+^ tissues revealed that osteopontin expression, in combination with CD44v6, correlates negatively with survival of patients with colorectal cancer^70^. Osteopontin also binds to cell surface αvβ3-integrin and activates tyrosine kinase signaling, thereby increasing CD44v6 protein synthesis while inhibiting its degradation^71^. This regulation occurs at the post-translational level without changes in mRNA expression. The increased CD44v6 maximizes the interaction with HA in the ECM and that HA adhesion is CD44-dependent via a competition with soluble HA. These findings suggest that the osteopontin/αvβ3-integrin/CD44v6 axis acts as an active signaling module that enhances HA-based adhesion and metastatic potential of liver cancer cells. Overall, the interaction between osteopontin and CD44 promotes cancer stemness, invasion, and immune suppression in several cancers including glioblastoma, colorectal cancer, and liver cancer.

#### Fibronectin

Fibronectin is a highly versatile cell adhesion glycoprotein that interacts with macromolecules such as fibrin, collagen, and proteoglycans as well as with cells expressing specific fibronectin receptors^72^. CD44 functionally cooperates with fibronectin at two different levels through direct binding to fibronectin^62^ and by regulating fibronectin expression during EMT and metastasis^73^.

Analysis of the binding levels of CD44 to different versions of fibronectin (full-length, cell-binding domain and C-terminal heparin-binding domain) showed that CD44 selectively bound to fragments that included the C-terminal heparin-binding domain of fibronectin^62^. This interaction contributes to leukocyte adhesion and trafficking. Changes in the cell-surface distribution of CD44 due to activation and conformational alterations increase its binding affinity for fibronectin^74^, suggesting that CD44 promotes lymphocyte adhesion to endothelial cells and migration to fibronectin-enriched inflammatory regions. In MDA-MB231 basal-like breast cancer cells, CD44 knockdown markedly reduced adhesion to fibronectin^73^. At the same time, the elevation of CD44 promoted strong adhesion to fibronectin-enriched matrices by upregulating α5β1 integrin, a canonical fibronectin receptor. Those results suggest that high CD44 levels correlate with increased adhesion to fibronectin, EMT, and invasion, partly via inside-out activation of fibronectin-binding integrins. Thus, CD44 can directly bind fibronectin through the C-terminal heparin-binding domain and indirectly potentiate fibronectin adhesion by upregulating α5β1 integrin during EMT.

#### Tumor necrosis factor-stimulated gene-6

TSG6, a secreted HA-binding protein, acts via its link domain and physically and functionally couples with CD44 to regulate the ECM structure and inflammatory response^10^. A structural study using wild-type TSG6 and link domain mutants identified five amino acids (Lys11, Tyr12, Tyr59, Phe70, and Tyr78) in the link domain of TSG6, which have important roles in HA binding^75^. The association of TSG6 with HA enhances its binding affinity for CD44 on the cell surface, suggesting that TSG6 has a significant effect on CD44-mediated cell activation at the inflammatory sites where it is expressed^63^. In addition, treatment with recombinant human TSG6 (rhTSG6) increased CD44 expression in A549 and HCC827 cells^60^. Immunoprecipitation assays in rhTSG6-treated human LUAD cells revealed that the interaction between TSG6 and CD44 promotes EMT and invasion^60^.

In hepatic stellate cells (HSCs), an immunofluorescence analysis showed that TSG6 and CD44 coexist on cell membranes^76^. TSG6 treatment increased the levels of inactive GSK-3β, a negative regulator of β-catenin, and stabilized β-catenin. Nuclear accumulation of β-catenin promoted the activation of yes-associated protein 1 (YAP-1), which drove stem-like transcriptional reprogramming in HSCs. Notably, CD44 knockdown using CD44 siRNA suppressed the activation of β-catenin and YAP-1 in HSCs, thereby inhibiting the conversion of HSCs into stem-like cells, indicating CD44 as a functional receptor for TSG6 (ref. ^76^). Therefore, TSG6/CD44/β-catenin/YAP-1 signaling pathway reprograms HSCs for fibrosis, with broader implications for EMT and metastasis in cancer contexts.

In several cancers including lung^60,77^ and colorectal cancer^78^, TSG6 is overexpressed and associated with poor prognosis. TSG6 promotes metastasis by stabilizing CD44 and enhancing CD44/EGFR complex formation^60,78^, which activates ERK signaling and the EMT in a CD44-dependent manner. These findings were generated by our group, which further demonstrated that TSG6 and CD44 are co-upregulated during transforming growth factor-beta (TGFβ)-induced EMT in A549 and HCC827 cells^60^. TSG6 also facilitates M2d-type TAM polarization in the tumor microenvironment through CD44-dependent Smad and MAPK pathways, linking TSG6/CD44 complexes to both cancer cell aggressiveness and immunosuppressive macrophage phenotypes. That study revealed that TSG6 acts as a scaffold that stabilizes CD44/TGFβR1/EGFR complexes^60^, thereby amplifying canonical TGFβ-Smad and EGFR-MAPK signaling during the EMT.

### CD44 as a signaling hub for RTKs via direct interaction

#### Epidermal growth factor receptor (EGFR) family

EGFR is physically and functionally connected to CD44 in various cancers^79–82^ (Table 3). Immunohistochemistry has demonstrated that CD44 interacts physically with EGFR in HNSCC^79^. More directly, immunoprecipitation and subcellular fractionation assay revealed that CD44 interacts with EGFR in non-small-cell lung cancer (NSCLC) cells treated with TSG6 (ref. ^60^). TSG6 treatment promotes the EMT and the polarization of TAMs by promoting the interaction between CD44 and EGFR and the activation of MAPK signaling. In addition, HA promotes the interaction between CD44 and EGFR, and binding between HA and CD44 enhances the EGFR-mediated signaling pathway and chemoresistance, as determined by EGFR inhibitor treatment^80^. Similarly, TGFβ1-dependent proliferation is mediated through the HA receptor CD44 by the interaction between CD44 and EGFR^83^. EGFR–CD44 coupling activates ERK1/2 as a proliferative response to TGFβ1. Therefore, the interaction between CD44 and EGFR regulates cell proliferation and malignant progression.

**Table 3:** Interacting receptor tyrosine kinases with CD44 for their activation.

Receptor tyrosine kinases	Mechanism	Refs.
Epidermal growth factor receptor (EGFR) (ErbB1)	Co-clustering of CD44 and EGFR inhibits degradation of EGFR, thereby stabilizing EGFR and sustaining EGFR-ERK signaling that supports tumor growth and drug resistance. CD44v6 functions as a co‑receptor for EGF-induced ErbB1 activation, which induces lymph node metastasis	^79–82,85^
HER2 (ErbB2)	Hyaluronic acid (HA)–CD44 interaction induces ErbB2 clustering and activation, thereby promoting proliferation, motility, and resistance to chemotherapy via PI3K–Akt and β‑catenin signaling pathways	^67,84^
ErbB3/4	CD44v6 functions as a co‑receptor for neuregulin-driven ErbB3 and ErbB4 activation, whereas CD44v3 acts as a co‑receptor for ErbB4, which activates ERK signaling	^85^
Vascular endothelial growth factor receptor (VEGFR)	CD44v6 acts as a co‑receptor for VEGFR2 that promotes cell migration, spheroid sprouting, tube formation, and tumor angiogenesis via ERK signaling pathway	^86,87^
c-MET (MET)	CD44v6 is required for complex formation with c‑Met, which enhances migration of endothelial cells and angiogenesis in tumors; blockade of v6 suppresses c-MET auto-phosphorylation and ERK signaling	^86^
Transforming growth factor-beta receptor type I (TGFβRI)	Binding of HA to CD44 stimulates TGFβRI kinase activity, increasing Smad2/Smad3 phosphorylation and promoting HA-mediated breast tumor cell migration	^60,83,88^
Platelet-derived growth factor receptor-beta (PDGFRβ)	HA‑dependent CD44 engagement increases complex formation with PDGFRβ and its phosphorylation, activating STAT3-Akt signaling to promote cancer stem cell traits	^67,89,153^
Fibroblast growth factor receptor 2 (FGFR2)	The physical interaction between CD44 and FGFR2 activates a main regulatory signaling pathway that drives stemness and tumorigenesis	^90,154^
Insulin-like growth factor 1 receptor (IGF1R)	HA–CD44 interaction enhances IGF1Rβ phosphorylation and co‑activates downstream PI3K–Akt and Src signaling pathways	^67,92,93^

Functionally, EGFR regulates CD44 expression, as treatment with EGFR inhibitor erlotinib downregulates CD44 levels in breast cancer^81^. Similarly, CD44 inhibition decreased EGFR signaling and increased the sensitivity of NSCLC having wild-type EGFR to cisplatin^82^. These data indicate that CD44 and EGFR mutually regulate each other in cancer progression and chemoresistance.

ErbB2, a member of the EGFR family, also interacts with CD44 in several breast cancer cell lines, particularly MCF7, MDA-MB231, MDA-MB435, and MDA-MB436 (ref. ^84^). CD44 consistently colocalizes and co-immunoprecipitates with both EGFR and ErbB2. In cytology samples from patients with metastatic breast cancer, CD44 colocalized with both EGFR and ErbB2 in ErbB2^+^ cases. However, in ErbB2^–^ samples (1 out of 16), it colocalized only with EGFR^84^. Together, these findings support the formation of a CD44–EGFR–ErbB2 complex in metastatic breast cancer.

CD44 variants are differentially recruited by ErbB ligands such as EGF^85^. CD44v6 was found to function as a co-receptor for EGF-induced and epiregulin-induced EGFR activation as well as for neuregulin-induced ErbB3 and ErbB4 activation in breast cancer cells, as determined by CD44v6 silencing or blocking peptide. CD44v3 isoform was required for pro-heparin-binding EGF-induced EGFR signaling. Therefore, CD44 variants are recruited for EGFR signaling in a ligand-dependent manner that promotes breast cancer cell survival and chemoresistance.

#### Vascular endothelial growth factor receptor (VEGFR) and mesenchymal epithelial transition (c-MET)

CD44 has been found to interact with VEGFR and c-MET (Table 3). In particular CD44v6 amplifies VEGFR2-mediated or c-MET-mediated angiogenic signaling^86^. Treatment with a v6 peptide mimicking the CD44v6 ectodomain or a v6-specific antibody inhibited the activation of VEGFR2 induced by VEGF-A or c-MET induced by HGF/SF, consequently suppressing the activation of ERK, their downstream factor. In addition, CD44v6 inhibition decreased endothelial cell migration, sprouting, and tubule formation. The C terminus of CD44 was found to bind to ezrin, linking it to the cytoskeleton in the activated state of VEGFR2 and c-MET. Blocking CD44v6 attenuated both invasion and endothelial cell responses triggered by VEGF and HGF. Those effects were also observed in an in vivo severe combined immunodeficiency (SCID) mouse xenograft tumor model with the CD44v6 peptide producing a significant reduction in tubular outgrowth and tumor-induced blood vessel formation in pancreatic tumor^86^. Therefore, VEGFR2 and c-MET are binding partners of CD44, especially CD44v6, in ways that affect angiogenic signaling in tumor progression.

CD44v6 is also required to bind VEGF and HGF, which functions as a co-receptor of VEGFR2 and c-MET, respectively, in several cancers and endothelial cells^87^. ELISA analyses revealed that both ligands bound specifically to CD44v6-expressing cells; but not to cells expressing the CD44s ectodomain. Additional pull-down assays, microscale thermophoresis, and fluorescence correlation spectroscopy analyses revealed their direct interaction with the CD44v6 ectodomain. When a purified CD44v6 ectodomain was used as a binding partner for VEGF and HGF, it disturbed the migration and scattering in a wounding assay, indicating that the purified exogenous CD44v6 ectodomain competed with endogenous CD44v6 to prevent the activation of downstream signaling including an ERK-mediated pathway. Thus, the study suggests that CD44v6 could be positioned as a potential drug target to inhibit pathological angiogenesis and invasive behaviors.

#### Transforming growth factor β receptor I (TGFβRI)

In several cancers, the TGFβ receptor, especially the TGFβ type I (TGFβRI) receptor, forms complexes with CD44 that affects the transcriptional regulation of TGFβ-mediated signaling (Table 3). In metastatic breast cancer cells, HA binding to CD44 induced an interaction between CD44 and TGFβRI receptor that enhanced TGFβ-Smad signaling and invasion^88^. Immunological data and Scatchard plot analyses showed that CD44 is physically linked to TGFβRI and TGFβRII and its cytoplasmic domain binds to TGFβRI at a single site. In addition, the binding of HA to CD44 stimulated TGFβRI kinase activity, which increased Smad2/Smad3 phosphorylation, leading to HA-mediated breast tumor cell migration.

In NSCLC, treatment with TSG6, a CD44 ligand, induced the EMT and polarization of TAMs^60^. In immunoprecipitation experiments, CD44 interacted with TGFβRI, and that was facilitated by TSG6 treatment. Those interactions triggered signals related to gene expression involved in the EMT and TAM polarization that amplified malignant progression. These results suggest that CD44 would also be important in TGF-β signaling of cancer progression.

#### Platelet-derived growth factor receptor (PDGFR)

PDGFR, especially PDGFRβ, physically interacts with CD44 in cancer (Table 3). In breast cancer, the CD44s interacts with PDGFRβ in an isoform-specific manner, inducing PDGFRβ phosphorylation and activating downstream STAT3-Akt signaling and thereby promoting CSC traits such as the formation of a mammosphere and tumor initiation^89^. Depletion of CD44s or overexpression of ESRP1, which inhibits the switch from CD44v to CD44s, reduced the activation of PDGFRβ-STAT3 signaling. Treatment with a PDGFR inhibitor or an STAT3 inhibitor blocked CD44s-induced CSC phenotypes. An analysis of TCGA data supported those patterns, showing that CD44s-associated genes have a strong positive correlation with the PDGFR pathway^89^. An additional study also showed that PDGFR-β activation enhanced cell survival through the PI3K–Akt pathway, with HA–CD44v6 acting as a co-receptor^67^.

#### Fibroblast growth factor receptor (FGFR)

In gastric cancer cells, CD44 promotes FGFR2 expression and activation, and FGFR2 also increases CD44 expression, maintaining gastric CSC traits through c-Myc as a common mediator^90^. In a co-immunoprecipitation analysis, the binding between FGFR2 and CD44 required both the membrane-proximal ECD and the ICD of CD44, suggesting a relatively direct interaction or the formation of a very tight complex. Depletion of CD44 and FGFR2 suppressed tumor sphere formation and affected their expression reciprocally. In addition, treatment with an FGFR2 inhibitor reduced the gastric CSC fraction of CD44^high^, whereas FGFR2 overexpression increased CD44 levels and accelerated tumor growth^90^. Therefore, the physical interaction between CD44 and FGFR2 activates a main regulatory signaling pathway for stemness and tumorigenesis.

#### Insulin-like growth factor 1 receptor (IGF1R)

A physical interaction between IGF1R and CD44 has not been broadly studied. Misra et al.^91^ reported that HA constitutively regulates the activation of RTKs in colon, prostate, and breast carcinomas. Immunoprecipitation experiments revealed that CD44 interacts with IGF1R-β to form a lipid-raft-integrated signaling complex. The interaction between HA and CD44 activates the association with IGF1R-β, which contains ezrin, PI3K, HSP90, and CDC37 and promotes resistance to apoptosis. In addition, HA/CD44/IGF1R complex enhances cell survival through the PI3K–Akt pathway, indicating that CD44 acts as a co-receptor for IGF1R-mediated tumor progression. When CD44 was blocked or HA was degraded, IGF1R was inactivated, suggesting that blocking the HA/CD44 complex would suppress IGF1R-mediated oncogenic signaling and progression^91^.

The interaction between CD44 and IGF1R mediates chemoresistance in hepatocellular carcinoma^92^. In sorafenib-resistant hepatocellular carcinoma cells, CD44 is highly expressed, which affects the activation of IGF1R and the formation of a CD44–GRP78–IGF1R signaling circuit that maintains drug resistance by being associated with the downregulation of TGFβ1. When TGFβ1/GRP78 was simultaneously depleted, CD44–IGF1R signaling was blocked, and sensitivity to sorafenib was restored, indicating that the CD44/IGF1R complex supports chemoresistance^93^.

Gemcitabine resistance in pancreatic cancer is also mediated by IGF1R-dependent CD44 upregulation^92^. Treatment with gemcitabine induced the phosphorylation of IGF1R and increased both the nuclear accumulation of c-Jun, ETS1, and EGR1 and their binding to the CD44 promoter region. Treatment with an IGF1R inhibitor (OSI-906) revealed that IGF1R regulates CD44 expression in gemcitabine-resistant pancreatic cancer cells. Silencing CD44 led to the recovery of E-cadherin levels, a reduction in zinc finger E-box binding homeobox 1 (ZEB1) expression, and an increase in drug sensitivity. Moreover, patients with pancreatic cancer with CD44^high^/IGF1R^high^ exhibited a poor prognosis^92^. Experimental and clinical data suggest that IGF1R and CD44 would be a good target for disabling metastasis and gemcitabine-induced drug resistance.

### CD44-mediated signaling and action mechanism in the cytoplasmic region

In CD44-mediated signaling, post-translational modification is important for triggering the signaling cascade in the cytoplasmic region. The CD44-ICD was generated by proteolytic cleavage and then translocated to the nucleus to bind to a specific DNA consensus sequence (CCTGCG) in the promoter regions of target genes^94^, involved in invasion, stemness, and drug resistance^94–96^.

When mammosphere-forming ability was compared with the levels of surface CD44 and CD44-ICD in six breast cancer cell lines, stemness correlated more strongly with high levels of CD44-ICD than those of surface CD44 (ref. ^95^). CD44 inhibition using siRNA reduced mammosphere formation and the expression of *NANOG*, *SOX2*, and *OCT4*. By contrast, overexpression of CD44-ICD restored sphere formation and stemness-factor expression even when surface CD44 was suppressed. Thus, CD44-ICD is an important regulator of stemness-related gene expression. To investigate whether CD44-ICD binds directly to the promoters of *NANOG*/*SOX2*/*OCT4*, γ-secretase inhibitors were treated^95^. Without γ-secretase inhibitors, CD44-ICD bound directly to *NANOG*/*SOX2*/*OCT4*, and they translocated to the nucleus together, as determined by immunoprecipitation and subcellular fractionation. When the cleavage was blocked, CD44-ICD remained in the cytoplasm, and the levels in the nuclear location decreased. Therefore, the nuclear translocation of CD44-ICD was deemed to be essential to the nuclear localization and stability of stemness factors. Using deletion mutants of the nuclear location signal or C-terminal PDZ (postsynaptic density protein 95, discs large, and Zonula occludens-1) domain of CD44-ICD, it was demonstrated that CD44-ICD is required to bind and activate the *SOX2*/*OCT4* promoter. Thus, the nuclear location signal and C-terminal PDZ domain of CD44-ICD are critical to the transcriptional activation of *SOX2*/*OCT4* for cancer stemness.

CD44-ICD also directly binds the *MMP9* promoter through an interaction with Runx2 and the CCTGCG sequences of its promoter in breast and ovarian cancer cells^94^. In cancer cells with high CD44 expression, the expression and activity of MMP9 increased in proportion to CD44 expression. CD44-ICD, generated through γ-secretase-dependent cleavage, translocated to the nucleus, associated with Runx2, and bound directly to the CIRE (CCTGCG) sequence in the *MMP9* promoter. Chromatin immunoprecipitation assay and electrophoretic mobility shift assay experiments revealed that CD44-ICD binds to the Runx2-adjacent CIRE to promote transcriptional activity, suggesting that the CD44-ICD/Runx2 complex regulates *MMP9* transcription^94^. Therefore, CD44-ICD functions as a transcriptional co-activator for cancer progression.

## Pathophysiology of CD44 in cancer

### Lung cancer

CD44 is frequently upregulated in NSCLC, especially in LUAD^97^. CD44 expression was investigated in 12 cell lines and 23 tissues of human NSCLC. In the LUAD samples, the mRNA and protein levels of CD44 were high in 5 of 5 cell lines and 10 of 14 tissues. By contrast, their expression was rare in lung squamous cell carcinoma: mRNA and protein expression of CD44 were detected in 1 of 6 cell lines and 0 of 9 tumor tissues^97^.

A meta-analysis was conducted to determine the effect of CD44 expression on survival in patients with NSCLC^98^. Those results showed that 55.3% of patients had high CD44 expression, and 42.5% had low expression. CD44 was highly expressed in 76.6% patients with lung squamous cell carcinoma and 31.9% patients with LUAD. Among patients with high CD44 expression, 37.5% had TMN stage I and II disease, and 27.6% had TMN stage III and IV disease. Compared with patients who had low CD44 expression, those with high CD44 expression had a positive lymph node metastasis rate of 49% (ref. ^98^).

To investigate the role of CD44 in NSCLC, CD44 was either overexpressed or inhibited^99^. When CD44 was overexpressed, cell proliferation and colony-forming ability were enhanced, compared with the control, as determined by CCK-8 and colony formation assays. By contrast, CD44 inhibition reduced cell proliferation and colony formation. Additionally, CD44 overexpression enhanced the migration and invasion of lung cancer by activating ERK-ZEB1 signaling^100^. An immunohistochemical analysis of lung cancer tissues demonstrated that primary tumors with elevated CD44 expression were associated with increased metastasis to regional lymph nodes. Consistently, a flow cytometric analysis revealed an enrichment of CD44-positive cells in metastatic lymph nodes, relative to the corresponding primary tumors. Furthermore, CD44 functions as a lung CSC marker because its expression was related to the stemness of lung cancer^101^. In one study, H1299 cells expressing CD44 showed stem-cell-like properties by expressing the pluripotency genes *OCT4*, *NANOG*, and *SOX2*.

The correlation between CD44 and immune cells was also investigated^102^. In LUAD, the correlation between CD44 expression and PD-L1 mRNA was analyzed using tumor immune estimation. This analysis revealed a positive correlation between CD44 and PD-L1 in patients with LUAD. Additionally, the correlation between CD44 and immune cells in patients with LUAD was analyzed using GSE103584, and a positive correlation was found between CD44 and B cells, CD8^+^ T cells, CD4^+^ T cells, macrophages, neutrophils, and dendritic cells^102^. Therefore, in lung cancer, CD44 functions as a stemness-associated receptor that promotes proliferation, metastasis, and immune evasion.

### HNSCC

CD44 mRNA and protein expressions were higher in HNSCC tissues than in normal tissues, according to an analysis of data from TCGA for 520 primary HNSCC and 44 normal tissues^103^. This analysis showed that high CD44 expression was associated with poor survival. Additionally, data for 2,102 patients with HNSCC were analyzed using open resources^104^. In patients with HNSCC overall, the expression rate of CD44 was 57.8%. Specifically, 66.4% of patients with pharyngeal cancer were CD44-positive, along with 54.7% of patients with laryngeal cancer and 49.3% of patients with oral cancer. No significant correlation was found between clinical features and CD44 expression in patients with oral cancer. However, CD44 was associated with advanced T stage (larynx: RR = 1.33, 95% CI 1.01–1.76; larynx and pharynx: RR = 1.21, 95% CI 1.08–1.35), worse N stage (larynx: RR = 2.53, 95% CI 1.99–3.21; larynx and pharynx: RR = 1.95, 95% CI 1.35–2.82), higher tumor grades (larynx and pharynx: RR = 1.71, 95% CI 1.04–2.79), and worse 5-year overall survival (larynx: RR = 0.62, 95% CI 0.47–0.83). In other words, CD44 was found to be associated with advanced T stage and N stage, higher tumor stages, and poor prognosis in pharyngeal and laryngeal cancers, but no clear association was observed between oral cancer and CD44 expression^104^. Among the CD44 variants, CD44v6 and v4 were highly expressed in HNSCC, as determined by quantitative reverse transcription–PCR, immunofluorescence, and flow cytometry. CD44v6 expression was highest in advanced metastatic HNSCC and tended to increase with tumor recurrence and metastasis^105^.

CD44 overexpression promoted the proliferation and migration of SCC25 cells^79^. Its expression was also associated with increased cisplatin resistance and apoptosis inhibition. CD44 and EGFR interacted in CAL27 cells, as determined by immunofluorescence and immunoprecipitation, and CD44 suppression inhibited EGFR phosphorylation and significantly reduced tumor growth in nude mice^79^. HA, a ligand of CD44, mediates the complexes formed by CD44 and EGFR to promote tumor survival and progression. MMPs were secreted by HA–CD44 signaling activation and were essential for tumor invasion^106^. LARG also binds directly to CD44 and EGFR, thereby activating Ras-mediated and RhoA-mediated signaling pathways that promote cell migration and tumor progression^106^. Therefore, CD44 is considered to be a critical factor for tumor progression, recurrence, and metastasis in HNSCC.

### Breast cancer

In breast cancer, CD44, especially CD44v6, is enriched in the aggressive, basal-like, and triple-negative subtypes, where it marks cancer stem-like cells, promotes invasion, and contributes to chemoresistance and poor prognosis^85,107,108^. High CD44 expression was associated with decreased overall survival and relapse-free progression in a Kaplan–Meier plot analysis of breast cancer patient survival. However, no relationship between CD44 and survival was found in the luminal subtype^107^.

An RNA-seq analysis of TCGA data compared gene expression differences between CD44s and CD44v^89^. The analysis of 802 genes related to CD44s and 356 genes related to CD44v revealed that CD44s correlated positively with CSC signature genes, whereas CD44v exhibited an inverse correlation. CD44s is predominantly expressed in breast CSCs, and CD44v did not contribute to tamoxifen resistance. That study also demonstrated that CD44s affects PDGFR signaling, but CD44v does not. CD44v inhibition did not show a relationship with PDGFR, but CD44s inhibition resulted in decreased PDGFR phosphorylation and reduced STAT3 phosphorylation, suggesting that CD44s–PDGFR–STAT3 signaling is involved in CSCs^89^. Therefore, alternative splicing provides distinct cancer cell states.

When CD44^+^CD24^–^ or CD44^+^CD24^low^ lineage cells were isolated from nine patients with breast cancer and transplanted intraperitoneally into NOD/SCID mice, tumors formed from a very tiny number of cells, approximately 100 cells in eight of the patient samples^11^. However, the CD44^–^ or CD24^+^ cells did not make tumors even when tens of thousands of cells were administered to the mice. Therefore, CD44 functions as a tumor-initiating inducer in the tumorigenesis of breast cancer^11^. CD44 expression also increases metastatic efficacy^109^. CD44 depletion using shRNA did not affect cell proliferation, but it significantly reduced invasion in Matrigel and adhesion to bone marrow endothelial cells. In mice injected with CD44-depleted cells, the median survival rates increased by ~20 days, and the systemic tumor burden, multiple metastases, and bone metastases were significantly reduced. Therefore, CD44 is a plausible target for treating metastasis in breast cancer.

In addition to CD44, HAS, a synthetic HA enzyme, has been studied as a target for breast cancer treatment. A comparison of HAS2 mRNA levels in MDA-MB-231 and LM2 breast cancer cells with CD44s overexpression or depletion found that CD44s and HAS2 levels are positively correlated^110^. CD44s promotes the expression of HAS2 via Akt signaling. Activation of Akt through CD44s leads to phosphorylation and inactivation of the transcription factor FOXO1, a transcriptional repressor of HAS2. This inactivation reduces its suppression, leading to increased HAS2 expression. The upregulated HAS2-dependent synthesis of HA further enhances CD44s–Akt signaling, maintaining the operation of a positive feedback loop. Therefore, targeting CD44s or HAS2 in combination with existing Akt inhibitors could have clinical significance by effectively suppressing cancer cell survival and therapeutic resistance, and targeting CD44 or functionally related factors such as HA or HAS could be a good anticancer strategy.

### Bladder cancer

CD44 expression on cell surfaces is related to aggressive phenotypes such as metastatic or recurrent bladder cancer. To analyze the correlation between CD44 expression and bladder cancer, 14 studies involving 1,107 tumor samples were analyzed^111^. Although the expression of CD44 in bladder cancer tissues was lower than that in non-tumor tissue samples (OR = 0.14, *P* = 0.005), CD44 expression showed a significant correlation with advanced T stage (OR = 1.76, *P* = 0.029) and lymph node metastasis (OR = 4.09, *P* < 0.001). In a multivariate survival analysis, CD44 expression was not associated with cancer-specific survival, overall survival, or recurrence-free survival, but it was significantly associated with disease progression and recurrence (HR = 2.912, 95% CI = 1.51–5.61), suggesting that CD44 expression might be related to aggressive cancer progression, metastasis, and recurrence in bladder cancer^111^.

When CD44v was observed in bladder cancer, CD44v3 had important roles in cancer progression, but CD44v6 did not^112^. When the expression of CD44v3 was suppressed using shRNA, factors related to tumor growth, such as p-Akt, p-ERK, and p-STAT3, were markedly downregulated. In addition, Bcl-2, an anti-apoptotic factor, decreased when CD44v3 was depleted in bladder cancer, indicating that CD44v3 not only contributes to tumor growth but also promotes anti-apoptotic properties. In addition, CD44v3 suppression induced cell arrest at the G0/G1 phase, which resulted in the deaths of those cells.

By contrast, CD44v6 depletion by shRNA did not cause significant changes in the levels of factors related to growth and anti-apoptosis effects^112^. The function of CD44v9 also showed significantly correlated relationship with malignancy in bladder cancer^113^. CD44v9 levels in 36 muscle-invasive bladder cancer (MIBC) tissues and 62 high-risk non-MIBC tissues were examined using immunohistochemistry. Twenty-five of the invasive bladder cancer samples (69.4%) showed low CD44v9 expression (*H*-score < 153), and 11 samples (30.6%) showed high expression. In this cancer type, the rates of lymphovascular invasion and lymph node metastasis were significantly higher in the CD44v9-positive group than in the negative group (64% versus 36%, 18% versus 8%). In high-risk non-invasive cancer tissues, 38 of the 62 samples (61.2%) showed low CD44v9 expression (*H*-score < 170), and 24 samples (38.8%) showed high expression. Patients with high CD44v9 expression had significantly lower progression-free survival and cancer-specific survival than those with low CD44v9 expression in both MIBC and high-risk non-MIBC samples. Therefore, the functions of CD44v isoforms differ depending on the cancer type and cellular context.

### Pancreatic cancer

To understand the correlation between CD44 expression and patient survival of pancreatic cancer, a Kaplan–Meier plot analysis was performed with 67 patients with pancreatic cancer^114^. The overall survival for CD44-negative patients was 28 months, whereas it was only 17 months for CD44-positive patients. Thus, CD44 expression is associated with poor prognosis in patients with pancreatic cancer. In a loss-of-function study using shRNA, CD44 depletion reduced clonogenic ability and mobility, compared with the control, in a Transwell cell migration assay, demonstrating that CD44 regulates cell migration and that its overexpression might contribute to pancreatic cancer metastasis^114^.

To understand its expression, CD44v6 was studied in 109 pancreatic cancer tissues^115^. The expression levels of CD44v6, CD133, and human tissue factor (TF) were all found to increase in pancreatic cancer. A clinical analysis revealed that individual expression of CD133, CD44v6, or TF was associated with vascular invasion, lymph node metastasis, and liver metastasis, and triple expression was significantly associated with lymph node metastasis. In univariate survival analyses, CD133, CD44v6, and TF were identified as risk factors for survival. The following multivariate analysis revealed that triple expression of CD133, CD44v6, and TF is an independent predictor of survival. Therefore, individual overexpression of CD133, CD44v6, or TF is related to pancreatic cancer metastasis, and the triple expression of these three molecules could serve as a useful marker for prognostication of pancreatic cancer^115^.

### Glioblastoma

CD44 is enriched in glioblastoma, the most aggressive brain tumor, compared with the levels in normal cortical tissue^116^. CD44 is involved in resistance to chemotherapy or radiotherapy^116^, hypoxic activity^117^, growth^118^, and invasion^119^ in glioblastoma. In a CD44 knockout study using CRISPR/Cas9 (ref. ^117^), the number of glioblastoma cells decreased when cell-cycle inhibitors such as p16^INK4a^ were upregulated. An RNA-seq analysis using those cells showed that the expression of hypoxia-related genes decreased when CD44 was suppressed. Therefore, in glioblastoma cells, CD44 is involved in proliferation and maintenance of hypoxic activity.

In an orthotopic glioma xenograft mouse model, CD44 depletion with shRNA suppressed glioblastoma growth and enhanced the sensitivity of glioblastoma cells to cytotoxic drugs^116^. CD44 is thus being explored as a therapeutic target in glioblastoma by using CD44-directed shRNA or CD44–Fc fusion proteins. When variant domains, including v3, v8, and v10, were tested, the heparin sulfate-modified CD44v3-v10-Fc fusion protein exhibited the most effective anti-glioblastoma effects^116^. HA-targeted agents such as 4-methylumbelliferone, which disrupts HA-CD44 signaling, reduced glioblastoma growth and migration^118^. Therefore, CD44 or related factors such as HA could be considered as promising therapeutic targets for glioblastoma treatment.

## Clinical implication and therapeutic development targeting CD44 and its ligand

CD44, a distinguished marker of CSCs, has been targeted in anticancer drug development, mainly in the form of monoclonal antibody (mAb) drugs, peptide drugs, and chimeric antigen receptor-T (CAR-T) cells. Early studies used antibodies such as RG7356, which binds to all CD44 isoforms, to target CD44s. However, recent attention has shifted to precisely targeting the specific CD44v forms expressed only in particular types of cancer. Moreover, drug-delivery strategies using HA, a ligand of CD44, as a carrier are also under development.

### Anti-CD44 antibodies

#### Unconjugated anti-CD44 humanized antibodies

Two anti-CD44 antibody drugs, RG7356 and bivatuzumab, have been developed and evaluated in phase I trials for solid tumors and acute myeloid leukemia (AML)^29,120–122^. RG7356, developed by Roche, is a humanized IgG1 mAb that binds near the HABD of all CD44 isoforms, including CD44s. It showed modest anticancer activity in phase I trials^120^. In AML, CD44 is an important target because it maintains leukemic stem cells. In a dose–response study of RG7356 in patients with relapsed or refractory AML, two cases of drug-induced aseptic meningitis occurred at the initial dose of 1,200 mg, but no further cases occurred after the premedication regime was strengthened^120^. During RG7356 treatment, CD68^+^ macrophages increased in the bone marrow, and CD34^+^ AML cells decreased, which aligns with preclinical results showing that RG7356 exerted antitumor effects through macrophage recruitment and phagocytosis.

It has been reported that RG7356 induces Fc-mediated macrophage activation and direct cytotoxicity in CD44^+^ cancer cells^121^. In a study evaluating pharmacokinetics and pharmacodynamics of RG7356 in 65 patients with advanced CD44-expressing solid tumors, CD14^+^ monocytes were found to transiently decrease depending on the dose and administration method, accompanied by a temporary increase in cytokines^121^. However, clinical efficacy was modest, and adverse effects such as fever, fatigue, headache, and abdominal pain occurred. The study was terminated early, but that was due to insufficient evidence of clinical and pharmacokinetic dose–response relationships, not safety issues^121^.

Bivatuzumab, previously known as BIWA4, is a humanized mAb against CD44v6, which is highly and homogeneously expressed in cancers derived from squamous epithelial cells, including in the head and neck, lung, skin, and esophagus. Because its expression is low in normal tissues, the different expression levels between normal tissues and tumor tissues make it a notable target for therapy^29^. A previous version of humanized bivatuzumab (BIWA4) is the VFF18 murine IgG1 mAb. In a screen of more than 500 different tumor samples, VFF18 showed the strongest and most homogeneous reactivity for squamous cell carcinomas, with limited reactivity in some epithelial cells and activated lymphocytes in normal tissues. When radiolabeled VFF18 was administered to an A431 epidermoid carcinoma xenograft model, rapid and selective tumor accumulation was observed within hours of administration, with a maximum tumor uptake of ~18% of the injected dose per gram of tissue^29^. The humanized antibody was then developed based on VFF18.

The epitope of these antibodies is the v6 domain on CD44v6. Bivatuzumab recognizes an epitope in the 360–370 amino acid region of the ECD of CD44v6. It binds to the sequence WFGNRWHEGYR as its core recognition site^29^. Bivatuzumab shows higher avidity for membrane-bound CD44v6 than the soluble forms, which improves tumor targeting^123^. Although internalization of the antibody after it binds to the antigen is less than 20% (ref. ^124^), its potential as a therapeutic target was proposed owing to the homogeneous expression of CD44v6 on tumor cell membranes.

#### Anti-CD44–drug conjugates

##### Bivatuzumab mertansine

Bivatuzumab has been developed into an antibody–drug conjugate (ADC) form by conjugating it with mertansine as a potent cytotoxic payload, thereby producing bivatuzumab mertansine (BIWI-1). Mertansine is an ultrapotent microtubule inhibitor originally derived from the natural product maytansine. It binds to tubulin to suppress microtubule dynamics and induces G2/M arrest and apoptosis^125^. Mertansine is associated with severe systemic toxicity when it is administered alone, limiting its clinical use, but it has been used in ADC strategies for tumor-selective delivery by linking it to antibodies through thiol-based conjugation sites^126^.

To evaluate its anticancer effect in CD44v6-positive tumors, a dose-escalation phase I clinical trial of BIWI-1 was conducted in patients with unresectable head and neck or esophageal squamous cell carcinoma. BIWI-1 showed low clearance (1.45 ml/min), a limited volume of distribution (5.25 l), and a half-life of 3–3.5 days, with an area under the curve increasing proportionally with dose across 25–325 mg/m^2^. At this dose range, no severe skin toxicity was observed, only mild adverse reactions such as fatigue, nausea, and vomiting were reported, and anti-drug–antibody responses were not detected^127,128^. Thus, at those doses, the concentration is likely insufficient for the toxic payload to accumulate in normal skin keratinocytes.

In a different study, BIWI-1 was administered intravenously once a week for 3 consecutive weeks, starting at a dose of 20 mg/m^2^ and increasing in 20 mg/m^2^ increments. Clinically, stable disease was observed in one patient each at doses of 100 mg/m^2^ and 120 mg/m^2^, accompanied by grade I skin toxicity^128^. At the highest dose of 140 mg/m^2^, one patient developed toxic epidermal necrolysis after two administrations, resulting in death due to extensive keratinocyte apoptosis in the skin^128^. On the basis of that case and skin-related adverse events repeatedly observed in four other phase I programs (70 patients across studies), the risk–benefit assessment led to the termination of its clinical development before the maximum tolerated dose was reached. The mechanism of skin toxicity observed with BIWI-1 is directly related to the expression of CD44v6 in normal tissues^128^. CD44v6 is expressed in basal and supra-basal keratinocytes in normal skin. When high doses of the ADC were administered, bivatuzumab bound to those cells, delivering the mertansine payload into them. Thus, microtubule disruption and apoptosis were induced, leading to widespread epidermal cell death and severe skin toxicity, including toxic epidermal necrolysis.

### H1D8-DC

H1D8 is a humanized IgG1 mAb designed to recognize the human CD44v5 epitope and specifically target the amino acid sequence IHHEHHEEEETPHSTST of CD44v5 (ref. ^129^). CD44v5, rather than CD44s, is highly expressed on the surface of intrahepatic cholangiocarcinoma (ICC), a type of liver cancer^130^. H1D8 was developed as an ADC called H1D8–DC (H1D8–drug conjugate) that comprises a humanized anti-CD44v5 antibody conjugated with a payload of monomethyl auristatin E (MMAE), a microtubule inhibitor. The ADC is conjugated via a valine–citrulline linker^131^. MMAE is a synthetic derivative of the peptide anticancer agent Dolastatin 10, isolated from the marine mollusk *Dolabella auricularia*^132^. It is a microtubule inhibitor that binds to tubulin and microtubules, disrupting the dynamic balance of polymerization and depolymerization, leading to G2/M arrest followed by apoptotic cell death^131^.

Compared with normal cells, the expression of CD44v5 is abnormally increased in ICC cells. CD44v5 expression is barely observed in most normal human organs, including normal skin tissue, so H1D8-DC might not be toxic to skin cells. As expected, the CD44v5-specific H1D8-DC showed potent and specific cytotoxicity in CD44v5-positive ICC cells^130^. In addition, it accumulated selectively in CD44v5-positive tumors in mouse models, inducing a strong tumor regression effect even in patient-derived xenograft models. Because its preclinical study in 2023 showed promising effects for ICC treatment, it is expected that clinical trials with H1D8-DC might be opened.

#### Radioisotope-labeled anti-CD44 antibodies

Because the antitumorigenic effects of anti-CD44v6 were moderate, the therapeutic modality was expanded to include a radioisotope-labeled antibody, as well as the previously tested ADC. In phase I radioimmunotherapy studies using the ¹⁸⁶Re-labeled chimeric mAb U36 and humanized bivatuzumab (BIWA4), repeated administration was possible^133^. Although tumor-absorbed doses varied depending on the lesion, ¹⁸⁶Re-bivatuzumab showed potential as an effective systemic adjuvant therapy for patients with head and neck cancer (HNC) harboring minimal residual disease. In addition, skin toxicity was rarely reported and mucositis was mild compared with prior external radiation therapy^133^. This favorable safety profile may reflect the β-emission characteristics of ¹⁸⁶Re, which has a relatively long half-life (about 89 h) and limited energy deposition, allowing a substantial proportion of radiation to dissipate beyond the thin epithelial layer where CD44v6 is expressed^128^. In the skin, in particular, part of the emitted radiation energy is released externally, so localized toxic effects similar to those seen with BIWI-1 did not occur^128,134^. Therefore, ¹⁸⁶Re-bivatuzumab shows a favorable balance between efficacy and tolerability in radioimmunotherapy for HNC.

^89^Zr-labeled RG7356 (anti-CD44 mAb) served as an immuno-positron-emission tomography (PET) imaging agent in a phase I study of patients with advanced and CD44^+^ HNC^121^. It was tested for tumor targeting but not used therapeutically. Tumor-targeting for PET using ^89^Zr-labeled RG7356 was observed for doses ≥200 mg. PET imaging can potentially be used in early drug development to confirm the expression of the drug target in tumor sites and to assess whole-body biodistribution over time. Thus, ^89^Zr-labeled RG7356 could be useful as a non-invasive PET imaging tool to validate CD44 expression and biodistribution.

### CAR-T cells

#### CD44-targeted CAR-T cells

CD44-targeted CAR-T development focussed on CD44v6 on CSCs in hematologic AML, multiple myeloma (MM), and solid tumors, including HNSCC, lung cancer, and gastric cancer^26,135–137^. The high expression of CD44v6 in AML and MM is associated with poor patient prognosis. Because it is not expressed in normal hematopoietic stem cells or progenitor cells^135–139^, CD44v6 has been proposed as a promising antigen for selective tumor targeting of CAR-T immunotherapy. On the basis of its expression profile, CD44 CAR-T products primarily target CD44v6 using second-generation or third-generation constructs derived from the BIWA8 mAb single-chain fragment variable, enabling MHC-independent recognition of surface CD44v6 on CSCs^140^. The mutation of *FLT3* or *DNMT3A* is frequently observed, with high levels of CD44v6 found in patients with AML, and relatively low CD44v6 expression in normal hematopoietic stem cells, suggesting the potential of CD44v6 as a safe CAR-T target. Humanized anti-CD44v6 single-chain fragment variable-based CAR-T cells induced selective cytotoxicity and high cytokine secretion against AML cells and primary cells with *FLT3* mutations^141^. Those data demonstrated strong anti-leukemia effects with acceptable toxicity in a xenograft mouse model.

Structure–function studies revealed that the splicing diversity of the CD44v6 antigen is an important factor in determining CAR spacer length^142^. CD44v6 is found in the membrane-proximal region, whereas the tumor-specific long isoform (v6–v10) exists in the membrane-distal region. Accordingly, CARs with short spacers (nerve growth factor receptor (NGFR) wild-type N variant 2 and mutated short; NWN2) more effectively recognized the proximal epitopes, whereas CARs with long spacers (NGFR wild-type long spacer; NWL) more effectively recognized the distal epitopes. In xenograft models of AML and ovarian cancer, CD44v6 CAR-T cells incorporating short spacers (NWN2) showed higher antitumor efficacy compared with the NWL construct. These data indicate that optimizing the spacer–epitope distance is critical for CAR function.

In preclinical studies, CD44v6 CAR-T cells demonstrated strong antitumor effects in AML and MM models without damaging hematopoietic stem cells or keratinocytes. In particular, in soft tissue sarcoma (STS) models, target recognition was possible in ~40% of CD44v6-expressing tumor cells, and specificity for killing was confirmed through antigen blockade experiments^143^. CD44v6-CAR⁺·cytokine-induced killer cells generated from patient-derived STS samples showed higher tumor-killing activity than conventional cytokine-induced killer cells, and they significantly inhibited tumor growth without causing toxicity in mouse xenograft models. Thus, CD44v6 is an antigen that functionally contributes to tumor growth in STS and is a viable immunotherapy target in solid tumors.

Despite the potent antitumor activity in preclinical models, CD44v6 CAR-T cells did not cause significant keratinocytes damage. This is partly attributed to the fact that the skin has a relative immune privilege, which is a physical factor that prevents NGFR-spaced CD44v6 CAR-T cells from penetrating and accumulating deeply into the epidermis. In addition, the activation of these CAR-T cells requires sufficient antigen density, co-stimulatory signals, and inflammatory cytokine environment. In contrast to the tumor environment, normal skin exhibits reduced inflammatory signaling and lower CD44v6 expression, thereby limiting full activation of CAR-T cells^144^ (NCT04097301).

The CAR-T cells targeting CD44v6 (MLM-CAR44.1) are autologous T cell preparations engineered via retrovirus to express CD44v6ΔNL CAR and the herpes simplex virus thymidine kinase (HSV-TK) Mut2 suicide gene. The suicide gene was introduced to minimize potential toxicity that could occur in normal CD44v6-expressing tissues (for example, skin, oral mucosa). These cells were evaluated for safety and efficacy in a first-in-human clinical trial (phase I–IIa) (NCT04097301) involving patients with relapsed or refractory AML or MM^139^. Although the clinical study was completed, the outcomes have not yet been clearly reported.

Overall, the rationale emphasizes CD44v6 as a promising target owing to its enrichment in CSC, its established role in metastasis, and its restricted expression profile. Specifically, its minimal presence on T cells and keratinocytes, when appropriately engineered, reduces the risks associated with pan-CD44 targeting.

#### Bi-specific or tri-specific CD44-targeted CAR-T cells

To enhance their efficacy and selectivity, CAR-T cells were generated using a bicistronic vector containing CD44v6-CAR and the HSV-TK suicide gene, and their efficacy was compared with CD19-CAR as a control^140^. In co-culture experiments with lung and ovarian cancer cells, the CD44v6-CAR-T cells induced a significant reduction in tumor cell viability. In non-obese diabetic IL2R^null^ SCID mouse xenograft models, the CAR-T therapy showed sustained in vivo expression, inhibited tumor growth, and significantly prolonged survival.

A tandem CAR-T cell targeting both CD44 and CD133 is being developed to treat recurrent glioblastoma (rGBM). rGBM currently has no established standard treatment, and the median survival period after treatment is less than 1 year. For this experiment, Tris-CAR-T cells were designed to simultaneously recognize CD44 and CD133 and incorporate a modified IL7Rα ICD to enhance T cell survival and functional persistence. CD44 and CD133 are known to be inversely correlated antigens in glioblastoma, so combining them as targets could reduce the selective survival of antigen-negative clones, which is a problem for single-antigen-targeted CAR-T therapy. In preclinical studies, Tris-CAR-T treatment induced long-term tumor suppression in intracranial xenograft models and glioblastoma patient-derived xenograft models without causing systemic toxicity or adverse effects^145^. In addition, when patient-derived autologous CAR-T cells were applied to glioblastoma organoid models derived from the same patients, strong antitumor activity was observed. On the basis of those results, CD44/CD133 dual-targeted Tris-CAR-T cells were administered to four patients with rGBM, and no neurotoxicity or adverse reactions exceeding grade 2 were observed. Moreover, within a few days after the first administration, radiological evaluations showed tumor shrinkage or growth inhibition, and improvements in patients’ clinical symptoms and neurological signs were also observed^145^ (NCT05577091). Therefore, multitargeting strategies would address antigen heterogeneity and escape by simultaneously engaging complementary tumor-associated antigens.

### Peptide inhibitor

#### AMC303

CD44 functions as a co-receptor for RTKs through direct interaction in signaling pathways in various cancers (see the section on Ligands of CD44). AMC303 (also known as HP558) is a cyclic 5-base polymer/oligonucleotide peptide inhibitor targeting CD44v6, developed by Hinova Pharmaceuticals Inc., under a license from Amcure. Preclinical studies suggest that it blocks the co-receptor function of CD44v6 rather than directly inhibiting the kinase domain of RTKs^86,146^, and that this activity depends on the functionally critical five-amino-acid motif (NEWQG) in CD44v6 (ref. ^146^).

Initial studies showed that CD44v6 is necessary for MET kinase activation and that a 5-base polymer/oligonucleotide peptide of the v6 region can inhibit MET-dependent cell migration, suggesting the possibility of disrupting the co-receptor function with a v6 peptide^146^. Later, it was found that v6 peptides interfere with the co-receptor function of CD44v6 and inhibit tumor growth and metastasis in pancreatic ductal adenocarcinoma models. Moreover, v6 peptides even induce the regression of metastatic lesions, indicating that the CD44v6–MET axis is associated with metastatic pancreatic ductal adenocarcinoma^30^.

The phase I/Ib results in patients with advanced CD44v6^+^ epithelial cancers (*n* = 55), including HNSCC, NSCLC, and cervical cancer, showed an excellent safety profile with no dose-limiting toxicities, favorable linear pharmacokinetics, and early antitumor activity with stable disease (NCT03009214). A phase II trial in patients with esophageal squamous cell carcinoma is ongoing in China.

#### A6 peptide

A6 peptide consists of eight amino acids (acetyl-KPSSPPEE-NH_2_) derived from the connecting peptide domain of urokinase plasminogen activator (uPA)^147^. Because binding of uPA to its receptor (uPAR) initiates tumor cell invasion and migration during metastasis, uPA–uPAR axis has been explored as a target for anticancer drug development. Interestingly, A6 shares sequences homology with the link domain of CD44 (residues 120–127; NASAPPEE)^148^, a region critical for HA binding.

Although A6 originates from uPA, it binds directly to CD44 without disrupting uPA–uPAR signaling. Instead, it induces conformational changes in CD44 that promote cell migration^147,148^. This interaction activates focal adhesion kinase and downstream MEK-ERK signaling in HA-dependent manner, ultimately sensitizing tumor cells to cell death. These effects have been demonstrated in animal models of ovarian cancer, breast cancer, and leukemia^147,148^. Clinical study with A6 was progressed through phase Ib with patients with ovarian cancer and phase II trial with persistent or recurrent ovarian epithelial cancer, fallopian tube cancer, or primary peritoneal carcinoma^149^ (Table 4). In a randomized placebo-controlled phase II trial, the patients received 150 mg of A6 via subcutaneous injection. The treatment was well tolerated, with no doses-limiting toxicities and favorable pharmacokinetic properties. However, objective antitumor activity was limited, and the proportion of patients achieving 6-month progression-free survival was low, leading to the conclusion that A6 was safe but showed minimal clinical efficacy. Exploratory analyses assessing the association between CD44 expression and clinical response were inconclusive, largely due to the limited samples’ number and variable CD44 expression^149^ (Table 4).

**Table 4:** Current status of clinical trials targeting CD44 in cancer.

Category	Therapeutic intervention	Cancer types	Delivery route	No. of patients	Phase and status	NCT no. (period)	Ref.
Pan CD44- antibody	RO5429083; RG7356	Advanced CD44^+^ solid tumors	Intravenous (IV) infusion	65	Phase I, completed	NCT01358903 (2011–2014)	^121^
CD44v6- antibody–drug conjugate (ADC)	Bivatuzumab mertansine	Head and neck squamous cell carcinoma (HNSCC)	IV infusion	31	Phase I, completed	NCT02254018 (2002–2005)	^155^
		Metastatic breast cancer		24	Phase I, completed	NCT02254005 (2002–2004)	^156^
Radiolabeled CD44v6 antibody	^99^mTc-labeled or ¹⁸⁶Re-labeled bivatuzumab	HNSCC	IV infusion	33	Phase I, completed	NCT02204033 (1999–2001)	–
	¹⁸⁶Re-labeled bivatuzumab	Breast adenocarcinoma		9	Phase I, completed	NCT02204046 (2000–2001)	–
	¹⁸⁶Re-labeled bivatuzumab	Non-small-cell lung cancer (NSCLC)		9	Phase I, completed	NCT02204059 (1999–2001)	–
CD44v6-targeting peptide	A6 peptide	Persistent or recurrent ovarian cancer, fallopian tube cancer, peritoneal carcinoma	Subcutaneous (SC)	31	Phase II, completed	NCT00939809 (2009–2012)	^149^
	AMC303 peptide inhibitor	Advanced or metastatic solid tumor	IV infusion	55	Phase I/Ib, completed	NCT03009214 (2016–2021)	^30^
CD44-targeting CAR-T cell	Genetically modified autologous T cells	Recurrent glioblastoma	Ommaya reservoir	10	Phase I, recruiting	NCT05577091 (2023–2032)	^157^

A phase II trial of A6 peptide for chronic lymphocytic leukemia was activated in 2014 posted by Angstrom Pharmaceuticals, Inc., but results remain limited^147^. Currently, the development of A6 peptide appears to have stalled after phase II. Phase III clinical trials have not been reported. Nonetheless, A6 continues to be used as a CD44-targeting moiety in preclinical nanomedicines^150^, reflecting its transformative value as a modulatory CD44 ligand rather than a single therapeutic agent. Therefore, A6 would be better as a CD44-targeting ligand for combinatorial or delivery-based strategies rather than a standalone therapeutic agent.

## Conclusion and future perspectives

The physiological roles of CD44 in cell adhesion, migration, and immune surveillance emphasize its evolutionary conservation. However, its pathological repurposing as a co-receptor for RTKs and CSC marker in various cancers reveals significant therapeutic vulnerabilities. Alternative splicing generates tumor-specific isoforms such as CD44v6 in squamous cell carcinoma, which allows for selective targeting. In addition, proteolytic cleavage of CD44 generates tumor-promoting CD44-ICD, which translocates to the nucleus and transcriptionally activates genes related to matrix remodeling such as *MMP9*, stem cell-associated genes including *SOX2*, and resistance factors such as ABC transporters.

Early pan-CD44-targeted approaches have shown limited clinical benefits, with RG7356 showing modest efficacy and bivatuzumab–mertansine producing skin toxicity. Those limitations have prompted a strategic shift toward isoform-specific interventions. Notably, the CD44v5-targeting ADC H1D8-DC, which is conjugated with MMAE, demonstrated reduced skin toxicity in preclinical ICC models. In addition, the CD44v6-targeting peptide AMC303 showed acceptable safety in phase I trials and has progressed toward phase II trials. In parallel, CD44v6-targeted CAR-T therapies are advancing through trials to treat both solid and hematologic malignancies.

Future therapeutic strategies are likely to emphasize rational combinations with RTK inhibitors or immune checkpoint inhibitors, biomarker-based patient selection, and innovative approaches to overcome the barriers of the tumor microenvironment and antigen heterogeneity. Overall, targeting CD44 is a powerful paradigm for disrupting CSC maintenance, metastatic spread, and drug resistance and has the potential to meaningfully improve clinical outcomes in CD44-driven malignancies.
